# Sea lampreys elicit strong transcriptomic responses in the lake trout liver during parasitism

**DOI:** 10.1186/s12864-016-2959-9

**Published:** 2016-08-24

**Authors:** Frederick Goetz, Sara E. Smith, Giles Goetz, Cheryl A. Murphy

**Affiliations:** 1 University of Wisconsin - Milwaukee, School of Freshwater Sciences, 600 East Greenfield Ave., Milwaukee, WI 53204 USA; 2 Michigan State University, Department of Fisheries and Wildlife, Lyman Briggs College, 480 Wilson Road 13 Natural Resources, East Lansing, MI 48823 USA; 3 NOAA Northwest Fisheries Science Center, 2725 Montlake Blvd., East Seattle, WA 98112-2097 USA; 4 NOAA Northwest Fisheries Science Center, Manchester Research Station, 7305 Beach Drive East, Port Orchard, WA 98366 USA; 5 Present Address: TerrAqua, Inc., 2989 Entiat River Rd, Entiat, WA 98822 USA

**Keywords:** Lake trout, Sea lamprey, Parasitism, Inflammation, RNA-seq, Differential transcription, Liver transcriptomics

## Abstract

**Background:**

The sea lamprey (*Petromyzon marinus*) is a jawless vertebrate that parasitizes fish as an adult and, with overfishing, was responsible for the decline in lake trout (*Salvelinus namaycush*) populations in the Great Lakes. While laboratory studies have looked at the rates of wounding on various fish hosts, there have been few investigations on the physiological effects of lamprey wounding on the host. In the current study, two morphotypes of lake trout, leans and siscowets, were parasitized in the laboratory by sea lampreys and the liver transcriptomes of parasitized and nonparasitized fish were analyzed by RNA-seq (DESeq2 and edgeR) to determine which genes and gene pathways (Ingenuity Pathway Analysis) were altered by lamprey parasitism.

**Results:**

Overall, genes encoding molecules involved in catalytic (e.g., enzymatic) and binding activities (factors and regulators) predominated the regulated gene lists. In siscowets, the top upregulated gene was *growth arrest and DNA-damage-inducible protein* and for leans it was *interleukin-18-binding protein.* In leans, the most significantly downregulated gene was *UDP-glucuronosyltransferase 2A2 -* DESeq2 or *phosphotriesterase related -* edgeR. For siscowets, the top downregulated gene was *C-C motif chemokine 19 -* DESeq2 or *GTP-binding protein Rhes* - edgeR. Gene pathways associated with inflammatory-related responses or factors (cytokines, chemokines, oxidative stress, apoptosis) were regulated following parasitism in both morphotypes. However, pathways related to energy metabolism (glycolysis, gluconeogenesis, lipolysis, lipogenesis) were also regulated. These pathways or the intensity or direction (up/downregulation) of regulation were different between leans and siscowets. Finally, one of the most significantly downregulated pathways in both leans and siscowets was the kynurenine (tryptophan degradation) pathway.

**Conclusions:**

The results indicate a strong transcriptional response in the lake trout to lamprey parasitism that entails genes involved in the regulation of inflammation and cellular damage. Responses to energy utilization as well as hydromineral balance also occurred indicating an adjustment in the host to energy demands and osmotic imbalances during parasitism. Given the role of the kynurenine pathway in promoting immunotolerance in mammals, the downregulation observed in this pathway during parasitism may signify an attempt by the host to inhibit any feedback suppression of the immune response to the lamprey.

**Electronic supplementary material:**

The online version of this article (doi:10.1186/s12864-016-2959-9) contains supplementary material, which is available to authorized users.

## Background

The sea lamprey (*Petromyzon marinus*) is a jawless fish that is native to the Atlantic Ocean. While it may also have been native to Lake Ontario [1], sea lampreys only became abundant in the Great Lakes following improvements to the Welland Canal that connects Lake Ontario to Lake Erie and bypasses Niagara Falls. Together with overfishing, the lamprey was responsible for the decline in lake trout populations in the Laurentian Great Lakes [2, 3]. As an adult, the sea lamprey is a parasite that attaches to fish with a rasping mouthpart and feeds off the tissues and body fluids of its host [4]. Significant efforts have been made to control lamprey in the Great Lakes and, while populations have been reduced, lamprey parasitism still remains an issue that could be exacerbated by global climatic changes affecting the Great Lakes [5]. The sea lamprey can parasitize a number of large bodied fish species, however, in the Great Lakes the effects on lake trout have been the most dramatic and have had the most significant consequences.

There have been a number of laboratory studies looking at the rates and types of wounding by lampreys on various fish hosts ([6] for review). In contrast, there have been surprisingly few studies that have investigated the physiology of the host during or following lamprey parasitism. For obvious reasons, mortality has been a major focus of research on lamprey parasitism. However, since many fish may survive lamprey wounding [7], it would be important to understand what occurs in the host during parasitism and how that could affect the physiology of the surviving host. Several investigators have looked at blood parameters after wounding and have shown increases in circulating lymphocytes [8, 9], and decreases [10] or increases [9] in blood hematocrit. Lampreys are parasites and foreign to their host, thus, it would be logical that the immune system of the host would react to the lamprey. However, to our knowledge there have been no investigations of the immune reaction of the host to lamprey parasitism. In other hematophagous parasites such as ticks, compounds are produced by the parasite that are released into the host to avoid host recognition or to block parts of the innate immune response (e.g., complement). This is thought to be strategic so that the host will not mount an immune response to the parasite (reviewed: [11]). We could hypothesize that similar activities might occur in a fish being parasitized by a lamprey.

Interestingly, there have been a number of studies that have isolated bioactive compounds from the buccal glands of other parasitic lampreys including *Lampetra japonica.* While a primary goal of those studies has been the isolation of compounds with potential pharmaceutical applications [12], they have uncovered several interesting compounds that may be important to the natural biological relationship of the lamprey and its host during parasitism. These include compounds that are active as inhibitors of lymphocyte proliferation, neutrophil activity and platelet aggregation [13–15], ion channel blockers [16], and compounds with fibrinolytic activity [13].

While a number of morphotypes of lake trout were once present in the Laurentian Great Lakes (e.g., [17]), only Lake Superior currently contains naturally sustaining populations of different lake trout types including the lean and siscowet lake trout. In the wild, siscowet lake trout morphotypes have larger fins and eyes, a shorter snout, larger caudal peduncle, and higher lipid content in the muscle than lean lake trout morphotypes [18, 19]. Lean lake trout tend to be distributed in waters shallower than 100 m while siscowet lake trout are found mostly at depths greater than 100 m [3]. In addition, lean and siscowet lake trout have different life histories with leans being shorter-lived, faster growing, maturing at a younger age, and experiencing higher mortality regimes [20, 21]. Studies have shown that some differences observed between wild siscowet and lean lake trout are likely to have a genetic or epigenetic basis [22, 23]. These include differences in growth and lipid levels in the muscle. In fact, it appears that leans and siscowets represent metabolotypes that can be distinguished by differences in energy reserves in the liver and muscle [23]. Given these differences in morphometry, physiology and life history, we were interested to see whether the response to lamprey parasitism would also differ between morphotypes. In the current study, lean and siscowet lake trout that have been reared in the hatchery from eggs to adults under identical environmental conditions [22] were used for controlled lamprey parasitism experiments in the lab. Endocrine and bioenergetic changes in relation to the lamprey parasitism on the hatchery-reared lake trout morphotypes have been presented separately [24]. Here we describe the changes in the hepatic transcriptome of lean and siscowet lake trout following lamprey parasitism. The results indicate a strong transcriptional response to lamprey parasitism that may involve reactions to an inflammatory and antigenic response brought on by lamprey wounding, and also suggest that there may be an interesting interaction of the lamprey with the immune system of the host. Responses to energy utilization as well as hydromineral balance were also observed, indicating an adjustment in the host to energy demands and osmotic imbalances that occur during parasitism.

## Results

### RNA-seq analysis

Across all 24 samples that were analyzed, there were on average 20,127,690 trimmed sequences/sample (Table 1, complete individual sequence data provided in Additional file 1). Of these, an average of 90 % mapped to the lake trout reference transcriptome produced by Trinity (all contigs provided in Additional file 2). When analyzed by DESeq2 and edgeR, there were 1341 and 668 genes regulated (up and down) between parasitized and nonparasitized leans at an adjusted *p* ≤ 0.05, respectively (Table 2, Additional files 3 & 4). Of these, a total of 452 genes were shared. In contrast, there were 2985 and 2343 genes that were regulated (up and down) between parasitized and nonparasitized siscowets at an adjusted *p* ≤ 0.05 when analyzed by DESeq2 and edgeR, respectively (Table 2, Additional files 5 & 6). Of these 1964 were shared.

**Table 1:** Average number of sequences for each treatment and the average number and percent mapped. Individual sample data are included in Additional file 1

Sample	Number	Raw counts	Trimmed counts	Mapped uniquely	Mapped ununiquely	Not mapped	Percent mapped
Leans parasitized	6	19,383,373	18,886,973	16,726,109	121,874	2,038,991	89
Siscowets parasitized	6	20,862,243	20,451,921	18,089,046	190,758	2,188,784	89
Leans nonparasitized	6	23,458,287	22,899,466	20,492,928	180,255	2,226,282	90
Siscowets nonparasitized	6	18,621,125	18,272,401	16,306,259	121,798	1,844,344	90
Average	24	20,581,257	20,127,690	17,903,585	153,671	2,074,600	90

**Table 2:** Number of genes regulated and shared between DESeq2 and edgeR analyses. Numbers of genes based on *p*adj values of ≤0.05 between parasitized and nonparasitized leans and siscowets

	Number of genes			
	DESeq2	edgeR	Shared	% Shared
Lean- parasitized/nonparasitized	1341	668	452	22
Siscowet- parasitized/nonparasitized	2985	2343	1964	37

GO annotation of the genes that were regulated by both the DESeq2 and edgeR analyses (intersection) indicates that the majority are involved in metabolic and cellular processes (Table 3). Based on the molecular function annotation, a majority of the genes encode molecules involved in catalytic (e.g., enzymatic) and binding activities (factors and regulators) (Table 4). In general, the percentage of genes involved in a given biological process did not differ when comparing genes up or down regulated during wounding (Table 3). However, in looking at molecular functions the proportion of several gene categories appeared to increase (e.g., receptor activity; catalytic activity) or decrease (e.g., translation regulator activity; enzyme regulator activity; transporter activity) when comparing up to down regulated genes, and this was consistent across morphotypes (Table 4).

**Table 3:** The proportion of genes that were shared between the DESeq2 and edgeR analyses (Table 2) within GO biological processes

Biological process	Leans up	Leans down	Siscowets up	Siscowets down
Cellular component organization or biogenesis (GO:0071840)	6.10 %	1.30 %	4.60 %	2.00 %
Cellular process (GO:0009987)	17.60 %	16.90 %	18.40 %	18.20 %
Localization (GO:0051179)	9.20 %	8.10 %	10.70 %	9.30 %
Apoptotic process (GO:0006915)	3.10 %	3.80 %	1.50 %	2.00 %
Reproduction (GO:0000003)	1.50 %	1.90 %	2.10 %	1.10 %
Biological regulation (GO:0065007)	9.20 %	8.10 %	10.10 %	9.50 %
Response to stimulus (GO:0050896)	6.90 %	10.00 %	6.30 %	8.60 %
Developmental process (GO:0032502)	6.10 %	7.50 %	6.90 %	5.60 %
Multicellular organismal process (GO:0032501)	3.10 %	4.40 %	5.40 %	4.10 %
Metabolic process (GO:0008152)	31.30 %	29.40 %	28.50 %	32.70 %
Immune system process (GO:0002376)	6.10 %	6.30 %	4.00 %	4.30 %
Locomotion (GO:0040011)	0 %	0.60 %	0.20 %	0.40 %
Biological adhesion (GO:0022610)	0 %	1.90 %	1.30 %	2.20 %

**Table 4:** The proportion of genes that were shared between the DESeq2 and edgeR analyses (Table 2) within GO molecular functions

Molecular function	Leans up	Leans down	Siscowets up	Siscowets down
Translation regulator activity (GO:0045182)	5.60 %	3.70 %	2.50 %	1.00 %
Nucleic acid binding transcription factor activity (GO:0001071)	2.80 %	2.40 %	4.30 %	4.80 %
Binding (GO:0005488)	25.00 %	25.60 %	27.70 %	24.70 %
Receptor activity (GO:0004872)	5.60 %	12.20 %	6.00 %	9.30 %
Enzyme regulator activity (GO:0030234)	4.20 %	2.40 %	7.10 %	4.20 %
Structural molecule activity (GO:0005198)	5.60 %	2.40 %	4.60 %	3.20 %
Catalytic activity (GO:0003824)	41.70 %	45.10 %	37.60 %	43.90 %
Transporter activity (GO:0005215)	9.70 %	6.10 %	9.90 %	8.00 %
Protein binding transcription factor activity (GO:0000988)	0.00 %	0.00 %	0.40 %	0.30 %
Antioxidant activity (GO:0016209)	0.00 %	0.00 %	0.00 %	0.60 %

Tables 5, 6, 7 and 8 show the top 25 up and downregulated genes for parasitized lean (Tables 5 & 6) and siscowet (Tables 7 & 8) lake trout. Of the top 25 upregulated genes based on adjusted *p* values, 16 were observed by both DESeq2 and edgeR between parasitized and nonparasitized siscowets (Table 7) but only five between parasitized and nonparasitized leans (Table 5). Within a RNA-seq analysis, one upregulated gene was shared between siscowets and leans for DESeq2 (Tables 5 & 7) though several other genes that appeared to have similar functions based on annotation (e.g., ubiquitin carboxyl-terminal hydrolase and ATP-binding cassette) were shared. There were three genes shared for edgeR (Tables 5 & 7). For siscowets, the top upregulated gene was *growth arrest and DNA-damage-inducible protein* (GADD45) in both RNA-seq and edgeR analyses, and for leans it was *interleukin-18-binding protein* (IL18BP) for both DESeq2 and edgeR (Tables 5 & 7).

**Table 5:**
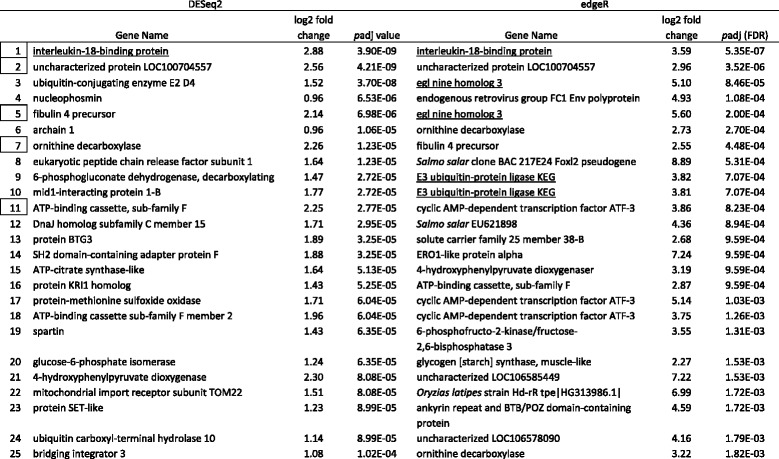
Top 25 annotated genes upregulated in parasitized versus nonparasitized lean lake trout. Genes ranked by *p*adj values. Boxed numbers indicate genes shared between the two analyses and underlined genes are shared between siscowets and leans within the DESeq2 and edgeR analyses. A complete listing of all genes is provided in Additional file 3 & Additional file 4. Note: There were no nonannotated genes in DESeq2 and edgeR in the top 25

**Table 6:**
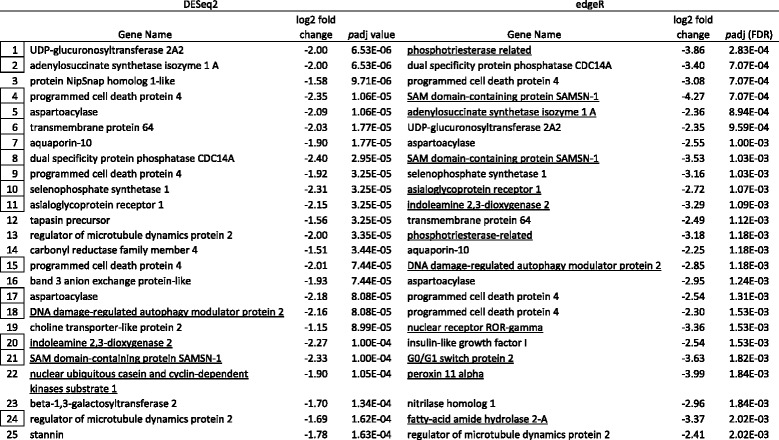
Top 25 annotated genes downregulated (− fold change) in parasitized versus nonparasitized lean lake trout. Genes ranked by *p*adj values. Boxed numbers indicate genes shared between the two analyses and underlined genes are shared between siscowets and leans within the DESeq2 and edgeR analyses. A complete listing of all genes is provided in Additional file 3 & Additional file 4. Note: There were 0 and 1 nonannotated genes in DESeq2 and edgeR, respectively in top 25

**Table 7:**
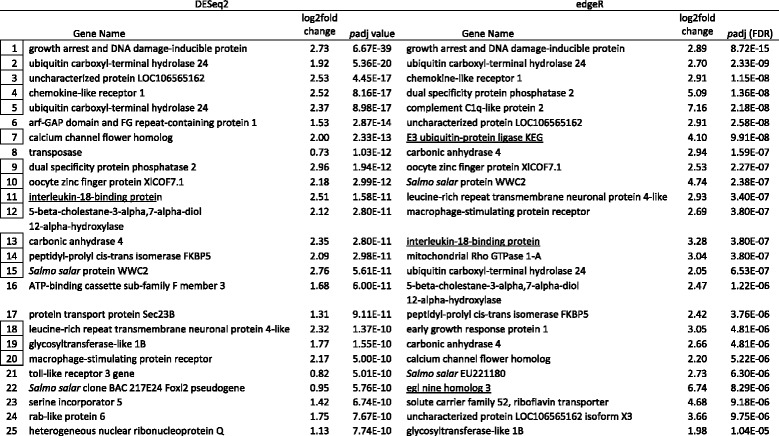
Top 25 annotated genes upregulated in parasitized versus nonparasitized siscowet lake trout. Genes ranked by *p*adj values. Boxed numbers indicate genes shared between the two analyses and underlined genes are shared between siscowets and leans within the DESeq2 and edgeR analyses. A complete listing of all genes is provided in Additional file 5 & Additional file 6. Note: There were one and three nonannotated genes in DESeq2 and edgeR, respectively in top 25

**Table 8:**
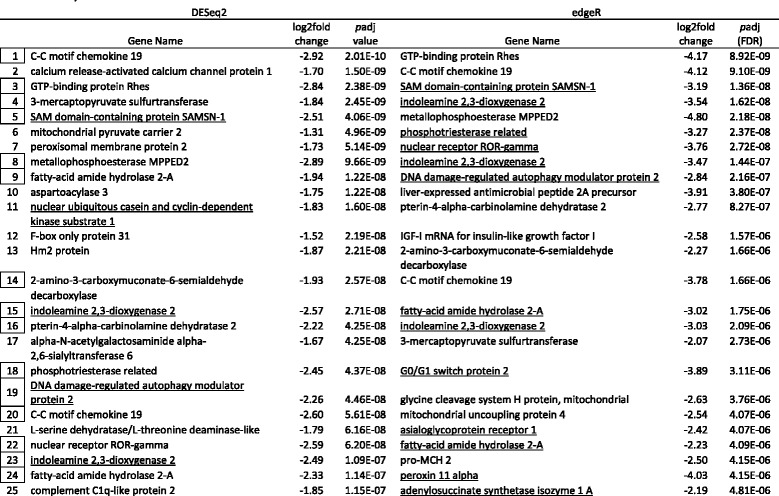
Top 25 annotated genes downregulated (− fold change) in parasitized versus nonparasitized siscowet lake trout. Genes ranked by padj values. Boxed numbers indicate genes shared between the two analyses and underlined genes are shared between siscowets and leans within the DESeq2 and edgeR analyses. A complete listing of all genes is provided in Additional file 5 & Additional file 6. Note: There were 0 and 1 nonannotated genes in DESeq2 and edgeR, respectively in top 25

Of the top 25 downregulated genes based on adjusted *p* values, 16 and 15 were observed by both DESeq2 and edgeR between parasitized and nonparasitized leans and siscowets, respectively (Tables 6 & 8). Within a RNA-seq analysis, four downregulated genes were shared between siscowets and leans for DESeq2 and 10 for edgeR (Table 6 & 8). In leans, the most significantly downregulated gene was *UDP-glucuronosytransferase 2A2* (UGT2A2) in the DESeq2 analysis or *phosphotriesterase related* (PTER) in the edgeR analysis (Table 6). UDP-glucuronosyltransferase 2A2 was observed in the edgeR analysis but *phosphotriesterase related* gene was not in the top 25 downregulated genes for leans though it did appear in the complete downregulated gene list (Additional file 4). For siscowets, the top downregulated gene was *C-C motif chemokine 19* (CCL19) when analyzed by DESeq2, or *GTP-binding protein Rhes* (Rasd2) when analyzed by edgeR (Table 8). The *C-C motif chemokine 19* was the second most significantly downregulated gene in edgeR, while the *GTP-binding protein Rhes* was the third most significantly downregulated gene in the DESeq2 analysis (Table 8).

### qPCR analysis

The results of qPCR analyses on at least five genes that were up or down regulated in either DESeq2 and/or edgeR analyses in leans and siscowets were highly consistent with the RNA-seq analyses (Table 9). In all cases, the direction of fold change (up or down) was exactly the same for all genes when comparing the qPCR analyses and the RNA-seq analyses. In addition, with a few exceptions, all of the qPCR comparisons (parasitized versus nonparasitized/morphotype) were significant at *p* < 0.05 or lower. Most of the ones that were not significant at *p* < 0.05, had nearly significant *p* values (e.g., 0.054, 0.070). In many cases trends in the overall magnitude of fold differences between the two analyses was also observed (Table 9; e.g., cyclic AMP-dependent transcription factor ATF-3 and dual specificity protein phosphatase 2).

**Table 9:** Results of QPCR analysis of genes appearing in the top 25 up and down-regulated gene lists for parasitized and nonparasitized lean and siscowet lake trout (From Tables 5, 6, 7 and 8). Contigs provided in Additional file 2

			QPCR		^a^RNA-seq
	Gene name	Contig #	Fold +/−	*p* value	Fold +/−
Lean	interleukin-18-binding protein	comp44889	26.4	0.004	9.51
	fibulin 4 precursor	comp81242	5.3	0.001	5.10
	glucose-6-phosphate isomerase	comp7122	3.2	0.004	2.36
	6-phosphogluconate dehydrogenase, decarboxylating	comp4673	3.4	0.005	2.77
	ornithine decarboxylase	comp4006	12.5	0.032	6.68
	cyclic AMP-dependent transcription factor ATF-3	comp224599	42.8	0.091	19.03
	indoleamine 2,3-dioxygenase 2	^b^comp228271	−5.2	0.007	
	indoleamine 2,3-dioxygenase 2	comp275449	−5.8	0.002	−6.87
	adenylosuccinate synthetase isozyme 1 A	comp122752	−4.1	0.001	−4.53
	UDP-glucuronosyltransferase 2A2	comp11363	−3.8	0.001	−4.53
	phosphotriesterase related	comp61354	−9.0	0.010	−14.52
	aspartoacylase	comp190057	−4.4	0.001	−4.99
Siscowet	growth arrest and DNA damage inducible protein	comp8599	7.5	0.012	7.01
	chemokine-like receptor 1	comp22172	3.3	0.038	6.59
	interleukin-18-binding protein	comp44889	6.8	0.054	7.46
	dual specificity protein phosphatase 2	comp204068	59.1	0.070	16.34
	ubiquitin carboxyl-terminal hydrolase 24	comp72729	2.9	0.004	4.96
	phosphotriesterase related	comp61354	−9.7	0.003	−7.26
	DNA damage-regulated autophagy modulator protein 2	comp92592	−7.0	0.008	−5.86
	indoleamine 2,3-dioxygenase 2	comp275449	−3.0	0.019	−5.62
	indoleamine 2,3-dioxygenase 2	comp228271	−5.7	0.001	−8.34
	GTP-binding protein Rhes	comp55581	−8.3	0.003	−11.39
	C-C motif chemokine 19	comp242068	−7.6	0.003	−11.47

^a^when present multiple times or in both DESeq2 and edgeR analyses, is the average fold change of all occurrences

^b^contig, comp228271, was not represented in parasitized leans but was still tested by QPCR

### IPA analysis

**Fig. 1:**
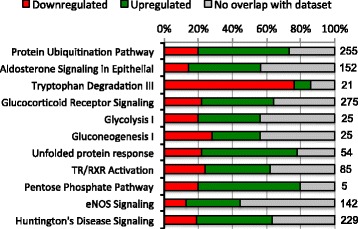
IPA pathways for which there was significant (*p* ≤ 0.05 Benjamini-Hochberg Method) overlap between genes from the edgeR analysis (*p*adj ≤0.05) and those in IPA for parasitized versus nonparasitized leans. Numbers at the right edge of the bars indicate total number of genes considered within each IPA pathway

**Fig. 2:**
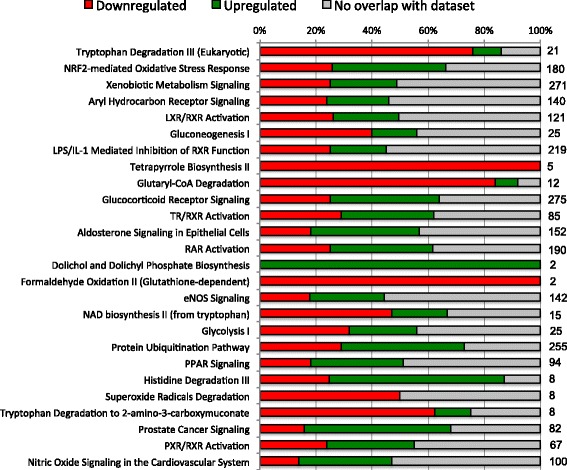
IPA pathways for which there was significant (*p* ≤ 0.05 Benjamini-Hochberg Method) overlap between genes from the edgeR analysis (*p*adj ≤0.05) and those in IPA for parasitized versus nonparasitized siscowets. Numbers at the right edge of the bars indicate total number of genes considered within each IPA pathway

**Fig. 3:**
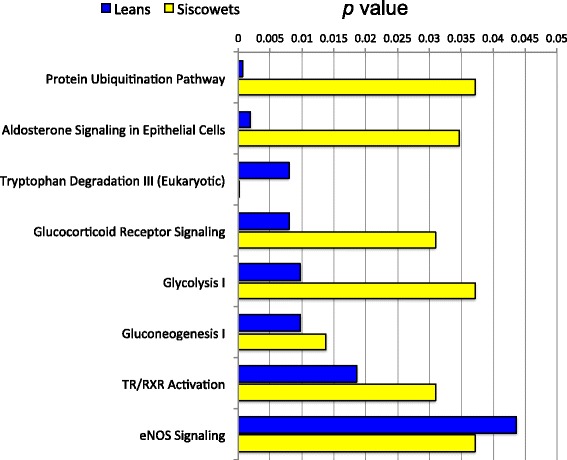
Significant (*p* ≤ 0.05 Benjamini-Hochberg Method) IPA pathways shared between parasitized leans and siscowets

IPA analysis showed that there was a total of 11 pathways for parasitized leans and 26 for siscowets in which genes from the edgeR analysis significantly (Benjamini-Hochberg Method; *p* ≤ 0.05) overlapped with genes in the IPA pathways (Figs. 1 & 2). For leans, the most significant (*p* ≤ 0.01) pathways were *protein ubiquitination, aldosterone signaling in epithelial cells, tryptophan degradation III, glucocorticoid receptor signaling, glycolysis I,* and *gluconeogenesis I* (Fig. 1, Additional file 7). For siscowets, the most significant (*p* ≤ 0.01) pathways were *tryptophan degradation III, NRF2-mediated oxidative stress response, xenobiotic metabolism signaling, aryl hydrocarbon receptor signaling*, and *LXR/RXR activation* (Fig. 2, Additional file 8). Of all the significant (*p* ≤ 0.05) pathways, eight were shared between leans and siscowets (Fig. 3).

## Discussion

The results of this study indicate that lamprey parasitism elicits a striking response in the hepatic transcriptome of both lean and siscowet lake trout. Some of these responses are shared between morphotypes but some are not. Since the fish were not perfused prior to tissue sampling, it is possible that some differences in gene expression could have been related to changes in the relative numbers of blood cells in parasitized versus nonparasitized fish as a result of wounding, but this would not affect changes in hepatic cell transcription. Overall, many of the genes that were in the regulated list were enzymes involved with catalytic processes. This is not surprising since the liver is the site of many enzymatic processes involving carbohydrate, lipid and amino acid metabolism and some of these processes appear to be affected by the wounding as discussed in detail below. In addition, genes that are involved with pathway regulation such as binding factors as well as the response to cell death (apoptosis) were also in the list.

As an adult, lampreys attach with a rasping mouthpart and feed off the tissues and body fluids of their host [4]. This dramatic wounding activity would be expected to have a significant impact on the physiology of the host yet this has not been well characterized in the literature. Increases in circulating lymphocytes particularly neutrophils [8, 9], and both decreases [10] and increases [9] in blood hematocrit have been reported. The wound that is produced during lamprey parasitism should have significant effects on the immune system of the host that could induce inflammatory and antigenic responses. Curiously, the transcriptomic response observed following wounding was not typical in comparison to what might be observed during pathogen (bacterial or viral) exposure. For example, cytokines like interleukins (IL) 1 and 6 or tumor necrosis factor (TNFα) were not in the regulated genes. It could be that these responses occurred at an earlier time just following parasitism and the sampling was completed after it. In support of this, genes (e.g., IL binding protein 18 - and see below for further details) that are believed to be anti-inflammatory and produced to help regulate inflammatory reactions, were in the regulated gene list. It could also be that the reaction brought on by the parasite wounding is fundamentally different from that of a pathogen since interactions with cellular components of the immune system in the host would occur with pathogens and elicit typical cytokine responses. Those interactions may not occur during lamprey wounding. Still, the RNA-seq analysis revealed the regulation of a number of genes in the liver following parasitism that could be a response to inflammation or tissue damage. For example, the most highly upregulated gene in siscowets was *growth arrest and DNA-damage-inducible protein* (GADD45), a gene that was originally characterized from cells that were subjected to agents such as UV, N-acetoxy-2-acetylaminofluorene and H_2_O_2_ that damage DNA [25, 26]. In humans there is a family of GADD45 proteins (α,β,γ) that are stress sensors upregulated under various physiological and environmental stressors. They are associated with cellular proteins that are implicated in cell cycle regulation and the response of cells to stress including PCNA (proliferating cell nuclear antigen), p21, cdc2/cyclinB1, and the p38 and JNK (c-Jun N-terminal kinases) stress response kinases [27–29]. From the wound produced by lampreys we might expect to see the stimulation of various proinflammatory cytokines in the host and a number of these including IL-6 have been shown to stimulate GADD45 proteins [30]. The reported outcome of GADD45 stimulation is complex and can be both cell protective or pro-apoptotic (cell death). To some extent this may depend upon the circumstance and/or the type of GADD45 protein being regulated [29]. How it may be functioning in the specific case of lamprey parasitism is unknown but the association of the regulation of this protein with wounding is logical given what is know about the function of these proteins. Very little has been published on GADD45 in fish though it has been proposed to be involved in demethylation and somatogenesis in zebrafish [31, 32] and it has been reported to be regulated in the liver of the Antarctic fish, *Trematomus bernacchii,* during heat stress [33]. Other genes that would logically be upregulated during stress such as the CCAAT/enhancer binding protein [34] were present in the gene lists for both leans and siscowets but were not significantly upregulated according to their adjusted *p* values. In contrast, heat shock proteins, also involved in cellular stress responses, were significantly (*p*adj < 0.05) upregulated in both leans and siscowets (DESeq2, Additional files 3 & 5) though they were not in the top 25 upregulated gene lists.

Significant upregulation of GADD45 was also observed in parasitized leans (*p*adj = 0.013; DESeq2, Additional file 3); though not in the top 25 upregulated genes. Instead, the most significantly upregulated gene in leans was the *interleukin-18-binding protein* (IL18BP) in both DESeq2 and edgeR analyses. This gene was also in the top 25 upregulated genes in parasitized siscowets. As indicated earlier, the wounding produced by the lamprey could produce an inflammatory reaction in the host and thus we could expect to see the expression of proinflammatory genes such as interleukins. Interleukin 18 (IL-18) has been identified in fish but the function is unclear [35]. In mammals, IL-18 is a cytokine that strongly stimulates interferon gamma. It is considered a proinflammatory cytokine but the actions are somewhat different compared to TNFα or IL-1 [36]. Interleukin 18 binding protein is an extracellular protein that has very high affinity for IL-18 and in mammals is believed to play a role in modulating the action of IL-18 given its strong activation of interferon [36]. If IL-18 has a similar function in fish, then the strong upregulation of the IL-18 binding protein may indicate that IL-18 is being upregulated in response to lamprey parasitism. It is important to note that IL18BP has homology to the interleukin 1 receptor, type 2 that is considered to be a decoy receptor for IL-1 [37] and this is upregulated in fish during LPS stimulation [38]. In addition, we did not find any upregulation of interferon. Thus, given the similarity in structure, it is unclear whether IL-1 or IL-18 binding protein is actually being regulated.

In contrast to the upregulated genes, there was less consensus between the DESeq2 and edgeR analyses for the top downregulated genes in siscowets or leans even though many downregulated genes were shared between the analyses. In siscowets, two genes, *C-C motif chemokine 19* (CCL19) and *GTP-binding protein Rhes* (Rasd2) were shared between the two analyses and were in the top three downregulated genes. In general, chemokines are leukocyte attractants that are involved during normal homeostasis and inflammatory conditions [37]. However, not much is known about chemokine 19 and, given the large number of chemokines, it is possible that the regulated sequence may actually be another structurally similar chemokine. GTP-binding protein Rhes is a GTP binding protein that is highly expressed in the mammalian brain and, in particular, the striatum [38]. However, it appears not to be expressed in the liver of mammals. Recently, Rasd2 has been shown to be an agent that activates autophagy in the brain [39]. Autophagy is a self-degradative process that can occur at different cellular levels and is involved in normal homeostasis and organelle and energy recycling, but can be ramped up during periods of cellular stress [40]. We could not find any reports on Rasd2 in fish but given the role in autophagy, upregulation of it may be relevant to degradative processes occurring during lamprey parasitism.

In leans, the top downregulated gene in the DESeq2 analysis was *UDP-glucuronosyltransferase 2A2* (UGT2A2) and in edgeR it was *phosphotriesterase related* (PTER) protein. UDP-glucuronosyltransferases are well studied enzymes that catalyze the formation of lipophilic glucuronides from substrates, including steroids, bile acids, bilirubin, hormones, dietary constituents, and thousands of xenobiotics using UDP-glucuronic acid as a cosubstrate [41]. As such they allow for solubilization and removal of lipophilic products that otherwise might be toxic to the body [41]. Glucuronidation has been frequently studied in the liver and involvement of this process is certainly consistent with the conditions occurring during lamprey parasitism where agents arising from inflammation or introduced into the host from the parasite (and see below) might be toxic. Therefore, why this gene would be downregulated rather than upregulated is unclear unless this was a result of some feedback activity to try and control this process. Phosphotriesterase related protein is more of an enigma since very little is known about the function of this protein in vertebrates. The PTER gene has been identified in mice, rats, humans and *Bombyx mori* [42, 43]. The precise role of this gene is unclear but in mice, silencing this gene using RNA interference diminished albuminuria-induced inflammatory and pro-fibrotic cytokine production in kidney tubular cells [43]. Thus, downregulation of this gene in the fish liver may be associated with the continued expression of inflammatory agents as a result of parasitism.

In this study we used and compared the results of two RNA-seq analyses; DESeq2 and edgeR. We were interested to see how consistent the results were across analyses and across morphotype. Compared to edgeR, DESeq2 found a greater number of regulated genes in parasitized leans and siscowets. When looking at genes downregulated during parasitism there was good agreement between the results of the two analyses within a morphotype with 16 and 15 of the top 25 genes shared for both leans and siscowets, respectively. That was also the case for the top upregulated genes in parasitized sicowets but not for leans where only five genes were shared between the two analyses in the top 25. Why this particular comparison did not show consistent results between analyses while others did is not clear. Interestingly the edgeR analysis for the top upregulated genes in parasitized leans had several occurrences of *cyclic AMP-dependent transcription factor ATF-3* (ATF3), a protein that is well characterized as being involved with cellular stress brought on by various stimuli including cytokines, genotoxic agents, apoptotic factors as well as conditions that promote amino acid and glucose deprivation [44, 45]. Given the inflammatory reaction and probable load on the host energy stores following parasitism, upregulation of this gene in the liver is logical.

That the RNA-seq analysis was accurately depicting the differential regulation of genes in parasitized versus nonparasitized lake trout livers was also confirmed by qPCR. All of the qPCR analyses indicated the correct direction of regulation and nearly all were significant when statistically analyzed.

While the analysis of regulated genes on an individual basis is interesting, a more global approach would be to look at the regulation of potential physiological or cellular pathways involving suites of regulated genes. We used IPA analysis to try and address this. Given the caveat that the pathways derived within IPA are based primarily on the proposed functions of their genes in mammals, this analysis indicated some interesting pathways that appeared to be regulated during lamprey parasitism in the liver. In this analysis, we employed a conservative approach using the edgeR RNA-seq gene analysis that had fewer genes overall than the DESeq2 analysis, together with the Benjamini-Hochberg Method to determine the significance of gene overlap with those of the IPA pathways. While we could have used the gene list from the intersection of the DESeq2 and edgeR analyses, we felt that some pathway information could be lost since those gene lists were greatly reduced compared with those from DESeq2 or edgeR. As observed with the number of individually regulated genes, there were more significant pathways uncovered with parasitized siscowets than leans. However, a number of these pathways were still shared between the morphotypes. In leans, the top functional pathway was *protein ubiquitination* and a majority of the genes in this pathway were upregulated. In the context of the IPA analysis, the protein ubiquitination pathway refers to gene products involved in the degradation of short-lived or regulatory proteins including ones in the cell cycle, cell proliferation, apoptosis, DNA repair, transcriptional regulation, cell surface receptors, ion channel regulators, and antigen presentation. All of these processes would logically be associated with lamprey parasitism particularly proteins involved in cell proliferation, cell cycle regulation and apoptosis given the strong upregulation of genes such as GADD45. While the protein ubiquitination pathway was also stimulated in siscowets this was not as significant as in leans (*p* = 0.001 lean vs 0.037 siscowet). In contrast, *tryptophan degradation* was the most significantly regulated pathway in siscowets but was also very highly regulated in leans (*p* = 0.008 lean vs 0.0001 siscowet). Tryptophan is an essential amino acid that can be a substrate for serotonin synthesis. However, when metabolized, approximately 95 % of tryptophan goes into the kynurenine (KYN) pathway [46]. The rate-limiting step in the KYN pathway is the enzyme that converts tryptophan to N-formylkynurenine. It is now known that at least three enzymes can do this: tryptophan 2,3-dioxygenase (TDO), indoleamine 2,3-dioxygenase-1 (IDO1) and indoleamine 2,3-dioxygenase-2 (IDO2) [47]. Studies have demonstrated that some fish species have genes for all three of these enzymes though efficiency for the conversion of tryptophan by the fish IDO2 enzyme is very low compared with mammals while IDO1 has moderate efficiency compared to mammals [48, 49]. In mammals, tryptophan 2,3-dioxygenase is found predominantly in the liver, while IDO1 and two are also found in the kidney and testes and less in the liver [47, 50]. Following the formation of kynurenines, there are two possible outcomes in the KYN pathway; a nonenzymatic conversion to quinolinic acid or conversion to 2-aminomuconic acid 6-semialdehyde by 2-amino 3-carboxymuconate 6-semialdehyde decarboxylase (ACSD) [51]. Interestingly, in both siscowets and leans the genes in the tryptophan degradation pathway were nearly all downregulated (Figs. 1 & 2) suggesting a strong inhibition of this pathway. In addition, in siscowets another pathway, *tryptophan degradation to 2-amino 3-carboxymuconate*, was also downregulated which would be the pathway catalyzed by ACSD. Consistent with these pathway observations, the genes for indoleamine 2,3 dioxygenase (IDO) as well as ACSD were consistently and significantly downregulated in the DESeq2 and edgeR analyses in both parasitized siscowets and leans (Tables 6 & 8; Additional files 3,4,5 and 6). Other pathways that were regulated according to the IPA analysis and are related to tryptophan metabolism include *glutaryl-CoA degradation* and *NAD biosynthesis II* (from tryptophan).

The KYN pathway has been strongly linked to immune function in mammals in various ways. For example: 1) Kynurenine metabolites produced in the KYN pathway can have direct effects on cells by the activation of the aryl hydrocarbon receptor; 2) Local depletion of tryptophan in a cell can activate a local stress response stimulating cell cycle kinases and transcription factors (like ATF-3 discussed earlier); and 3) IDO can, in addition to being an enzyme, act directly as a cellular signaling molecule [52]. The net result of KYN activation is complex and can involve many inputs. In mammals the KYN pathway is stimulated during inflammatory reactions via interferons but the kynurenine metabolites produced may ultimately function as immunsuppressors [52]. Indeed, it is known that IDO stimulation promotes immunotolerance of grafted allogeneic tissues whereas inhibition of IDO results in rejection [53]. So one hypothesis is that the stimulation of IDO results in dampening of the immune response and immunotolerance. Thus, predicting the overall immune response of downregulating or upregulating this pathway is difficult particularly since it is unknown if there are similar IDO functions in fish. As far as we can tell, the relationship of the KYN pathway and immunity has not been investigated in any fish species though transcripts encoding IDO2 were downregulated in rainbow trout fry following challenge with *Flavobacterium psychrophilum* [54]. It seems clear that this pathway is downregulated following lamprey parasitism and if the KYN pathway is ultimately immunosuppressive in fish and acts to temper the inflammatory reaction, then downregulation might be a mechanism to block immunosuppression and continue to respond to the presence of the lamprey (i.e., not be immunotolerant).

The KYN pathway has been extensively investigated during infections by intracellular parasitic protozoans such as *Leishmania major* [55]. During leishmaniasis the KYN pathway is stimulated resulting in local depletion of tryptophan and kynurenine production. In gene knockout mice lacking IDO or folIowing the application of IDO inhibitors, there is actually a decrease in *Leismania* infection suggesting that pathogens such as *Leishmania* may act to suppress the host immune system by stimulating the KYN pathway and thereby promoting immunotolerance [55]. In other parasitic lampreys (*Lampetra japonica*) a number of products have been isolated from the buccal gland [12] that are probably released around the wound site and into the host circulation. As with other hematophagous parasites, some of these compounds are probably released to keep blood from coagulating so that feeding of the circulation by the lamprey can continue. Indeed, experiments conducted some time ago on the sea lamprey demonstrated that fluid obtained directly from the buccal glands inhibited clotting of fish blood [4]. At the same time it was found that injection of small volumes of sea lamprey buccal gland secretion into the muscle of nonparasitized fish caused the formation of very large edemas suggesting the presence of compounds that could be highly cytolytic. Some compounds in the buccal gland secretions may be released in an attempt to block the immune response of the host or be used to hide from the host. Curiously, L-3-hydroxykynurenine O-sulfate has been isolated from the buccal glands of the parasitic lamprey, *Lethenteron japonicum* [56]. Could this kynurenine be released by the lamprey into the circulation of the fish host and act to mimic the stimulation of the host's KYN system? If so, this may be a mechanism that the parasite uses to promote immunosuppression so that it can continue to parasitize the host. In any case, compounds (particularly proteins) that are produced by the lamprey and released into the circulation during parasitism may add to the overall antigenic response occurring within the host and be responsible for some of the pathways being simulated.

Two carbohydrate bioenergetic pathways that were regulated were *glycolysis* and *gluconeogenesis*. These were regulated significantly in both leans and siscowets but glycolysis was regulated to a greater extent in leans than siscowets (*p* = 0.01 leans vs 0.04 siscowets). Glycolysis is the process in which glucose is metabolized to pyruvate and results in the production of ATP. Gluconeogenesis is the reverse of glycolysis and hence the production of glucose. While most of the reactions in glycolysis are reversible there are some differences primarily as a result of the steps in which energy is produced and these include the conversion of pyruvate to phosphoenolpyruvate, fructose 1,6-bisphosphate to fructose 6-phosphate, and glucose 6-phosphate to glucose. While not dramatic, another difference between leans and siscowets with regard to glycolysis was that in leans it appeared that there was a greater proportion of genes upregulated (Fig. 1) while in siscowets it was almost equal or even slightly more downregulated (Fig. 2). In the wild, siscowets have higher muscle lipid than leans and this is a heritable trait [22]. In fact, leans and siscowets can be considered metabolotypes in which a number of energetic characteristics are different between them including lipid (higher in muscle and liver in siscowets vs leans) and glycogen (higher in muscle and liver in leans vs siscowets) [23]. Given the consumption of host tissue and blood, lamprey parasitism must be bioenergetically draining, and how the host compensates for that most likely depends on the way energy is stored. Given the differences in lipid and carbohydrate between the two morphotypes, it may not be surprising that glycolysis is upregulated in leans to a greater extent than in siscowets. In addition, in siscowets several IPA pathways involved in lipid metabolism or the regulation of lipid metabolism were regulated including *LXR/RXR (liver X receptor/retinoid X receptor) activation*, *PPAR (peroxisome proliferator-activated receptor) signaling*, and *PXR/RXR (pregnane X receptor/retinoid X receptor) activation* [57]. None of these pathways were observed to be regulated in parasitized leans. It appears that in the wild, siscowets are parasitized at a higher rate and more intensely than leans [24]. While there could be several reasons for this difference, the high lipid levels in siscowets may make them more capable energetically of sustaining lamprey parasitism events.

Two other pathways that were significantly regulated in both leans and siscowets were *aldosterone signaling in epithelial cells* and *glucocorticoid receptor signaling* and these may be related. Based on *p* values these two pathways appeared to be more highly regulated in leans than siscowets and for both morphotypes there was a greater proportion of genes that were upregulated. In the case of IPA the aldosterone signaling in epithelial cells involves genes of the phosphatidylinositol and protein kinase C intracellular signaling pathways as well as Na^+^/K^+^ATPase pumps and channels. The glucocorticoid receptor signaling involves some similar second messenger pathway genes but also genes involved in inflammation and cell cycle control. Both of these pathways are logical given the possible inflammation associated with the parasitism and since there would likely be ionic/osmotic imbalances during the wounding, pathways involving ion pumps and channels would be logical. The cortisol stress response has been well documented in fish [58] and it is likely that lake trout experiencing lamprey parasitism undergo stimulation of the hypothalamic pituitary interrenal axis that would result in elevated cortisol and the stimulation of the glucocorticoid receptor pathway. Whether aldosterone really exists in fish is debated [59] and the mineralcorticoid in fish may be other steroids. However, cortisol also functions as a mineralcorticoid in fish and, thus, the pathways designated as those specific to aldosterone in the IPA analysis could in effect be stimulated by cortisol, particularly those that regulate Na^+^/K^+^ ATPase [59].

A major pathway that was significantly regulated in siscowets (*p* = 0.0003) but not in leans (*p* = 0.1208) was *NRF2-mediated oxidative stress response*. In IPA this pathway involves gene products that are regulated by the nuclear factor-erythroid 2-related factor 2 (NFE2L2) in response to oxidative stress caused by an imbalance between the production of reactive oxygen and the detoxification of reactive intermediates by enzymes including glutathione S-transferase, cytochrome P450, NAD(P) H:quinone oxidoreductase, heme oxygenase and superoxide dismutase. Many things can cause oxidative stress but certainly inflammation is one of them and so it is not surprising to see this pathway activated during parasitism. NFE2L2 regulates many enzymes known to be involved in the detoxification of drugs and chemicals that are foreign to the body [60] so we might expect to see associated pathways such as the *xenobiotic metabolism signaling* also being significantly regulated. NFE2L2 can also influence intermediary metabolism and has been show to regulate AhR (aryl hydrocarbon), PPAR, and RXR receptors that contain ARE (antioxidant response element) sites [60]. So again, is not surprising to see those pathways (*aryl hydrocarbon receptor signaling, PXR/RXR activation, PPAR signaling*) being regulated and, if this is related to lipid metabolism, may explain why the *NRF2-mediated oxidative stress response* was regulated in siscowets and not leans.

## Conclusion

In conclusion, it appears clear from the RNA-seq analysis that during lamprey parasitism, there is a very strong response in the liver that entails genes involved in the regulation of inflammation and cellular damage. In some cases it looks like genes may be stimulated as a feedback mechanism to the responses being mounted in the host. Overall, the IPA analysis indicates the involvement of pathways related to 1) energy metabolism (glycolysis, gluconeogenesis, lipolysis, lipogenesis); 2) removal and degradation of molecules arising from cellular processes such as apoptosis and oxidative stress; 3) hydromineral balance and 4) tryptophan degradation (KYN pathway). In fact, several pathways related to tryptophan degradation were observed and we hypothesize that these are actually responses to immune reactivity brought on by the lamprey wounding and may even involve compounds produced by the parasite that are released into the host. Several of these pathways including tryptophan degradation, hydromineral balance, and ubiquination were shared by both morphotypes but there were also noticeable differences particularly in pathways related to carbohydrate and lipid metabolism. There are very large natural differences between leans and siscowets in the levels of carbohydrate and lipid reserves and, therefore, differences observed in these metabolic pathways may depend on these energy reserves and have biological relevance in terms of how the two morphotypes cope energetically with lamprey parasitism.

## Methods

### Animals and lamprey parasitism trials

Lean and siscowet lake trout used for the laboratory lamprey parasitism were part of a common garden rearing study investigating the basis of phenotypic differentiation of these morphotypes that was previously described [22]. Briefly, the original lean and siscowet laboratory lines were derived from gametes of wild adult fish obtained in 2006 from Lake Superior. The fertilized eggs and subsequent juveniles and adults were reared under identical environmental conditions from 2006 at the Great Lakes WATER Institute (GLWI, School of Freshwater Sciences, University of Wisconsin-Milwaukee). Lamprey parasitism experiments for the transcriptomic experiments were conducted from October through December 2010 when sea lamprey seasonally intensify their feeding to prepare for spawning [61]. Sea lamprey were obtained from commercial fisherman in the Hammond Bay, Michigan and Blind River, Ontario areas and transported to our facilities. All sea lamprey were parasitizing a host at the time of capture to ensure that the sea lamprey used in this experiment were in the parasitic phase.

Lake trout were anesthetized individually in 2-phenoxyethanol (Sigma-Aldrich, St. Louis, MO), weighed, and placed in individual covered tanks (265 L) for experimental trials. Each test lake trout was randomly paired with a control lake trout of the same morphotype that remained in its individual tank for the same duration of time but was not parasitized. Test and control lake trout were usually of the same sex, although errors in sex identification did occasionally occur, as lake trout are not obviously sexually dimorphic. The lake trout used for these trials were four years old and not sexually mature. Sea lampreys (*N* = 4) were randomly chosen, weighed, identified by fin clips, and placed in each test lake trout tank. After the addition of sea lamprey to the test tanks, test and control lake trout were checked three times per day at regular intervals. Once a sea lamprey attached to a lake trout, the other non-attached lampreys were removed from the tank. We estimated sea lamprey feeding duration to be the period from when the sea lamprey was first noted to be attached to when the sea lamprey was first noticed to have detached or was physically detached from the test lake trout. The average parasitism time was 3.2 days for both morphotypes and ranged from 2 to 4 days. After the experimental trial, test and control lake trout were euthanized using an overdose of tricaine methanesulfonate (MS-222) (Sigma-Aldrich, St. Louis, MO). Lake trout and sea lamprey final weights were recorded to aid standardization of parasitism events. The number and type of sea lamprey wounds on the lake trout were characterized and blood and gonad samples were taken for physiological analyses described separately [24]. A liver sample was taken from each fish and flash frozen on dry ice and stored at −80 °C until RNA extraction. All experiments were performed in strict accordance with Michigan State University’s Institutional Animal Care and Use Committee (IACUC) approved procedures.

### Transcriptomic analysis

Total RNA from six liver samples/treatment/morphotype (lean nonparasitized; lean parasitized; siscowet nonparasitized; siscowet parasitized) was extracted on an individual basis using Tri Reagent (Molecular Research Center, Inc.) according to the manufacturer’s protocol [62, 63]. The RNA was treated with DNAse I and cleaned using the RNeasy MinElute Cleanup kit (Qiagen, Valencia, CA) and submitted to the University of Washington High Throughput Genomics Unit at the University of Washington (Seattle, WA) for sequencing. Individual libraries were constructed using the TruSeq RNA library kit (Illumina) and sequenced (36 bp single end) using the Illumina GAIIx platform (San Diego, CA). Sequences were barcoded and all 24 samples were sequenced in the same lane and this was repeated on different dates for a total of three lanes. For transcriptomic analysis, sequences were combined across all three lanes for each treatment (parasitized vs nonparasitized) per morphotype (lean vs siscowet). All raw sequences are available at NCBI's Sequence Read Archive (SRA) under Project PRJNA316738.

Sequences were trimmed for quality (cutoff 0.05) using CLC Genomics Workbench (6.5.1), ends were trimmed for ambiguous bases, and adapters (Illumina) were removed. Sequences less than 20 bp were removed. Sequences from the individual samples were combined with sequences that had been obtained previously from a preliminary pooled experiment on the same samples to produce a *de novo* assembly using Trinity version r2013-02-25 with default settings [64]. The assembled contigs (42,077, average 577 bp, median 356 bp, Additional file 2) were then annotated using BLAST and NCBI’s nr and nt databases [65–67]. Individual sequences were mapped to the *de novo* assembled contigs using CLC Genomics Workbench. Count data for each sample’s run were totaled into a single table for each sample. The count data were then analyzed for gene expression levels and statistical significance using the following R packages: DESeq2 [68] and edgeR [69]. Within the text, gene names are initially italicized when referred to and, when available, the HGNC (http://www.genenames.org/) accepted symbol is provided in parentheses. Genes (up and down regulated/lean and siscowet) that were shared between the DESeq2 and edgeR analyses were GO annotated at the biological process and molecular function levels using Panther [70] that accesses the most up to date GO annotations at the Gene Ontology Consortium.

### Quantitative Polymerase Chain Reaction (qPCR) analysis

Complimentary DNA (cDNA) was produced by reverse transcription in a PTC200 thermocycler (Bio-rad MJ Research). Oligo(dt) primer (0.25 μg) was added to 500 ng of total RNA in a volume of 5 μl. The mixture was allowed to incubate at 70 °C for 5 min, and then 4 °C for 5 min. Following this, 4 μl of 5× reaction buffer, 2.4 μl of MgCl_2_ (25 mM), 1 μl of dNTP mix (10 mM), 1 μl of Promega ImpromII RT, and 6.6 μl of water were added and incubated at 25 °C for 5 min, 37 °C for 1 h, and 70 °C for 15 min.

All qPCR reactions were created as master mixes and individual reactions were conducted in duplicate and contained the following: 1.0 μL of cDNA, 10 pM each of forward and reverse gene primers (Additional file 9), and 10 μl Lightcycler 480 SYBR Green PCR Master Mix (Roche). Cycling and fluorescence measurements were carried out in a Lightcycler 480 II qPCR system (Roche) with the following cycling parameters: 1 cycle of 95 °C for 5 min; 45 cycles of 95 °C for 10 s, 58 °C for 10 s, and 72° for 10 s.

Raw data were processed with Real-time PCR Miner [71]. Quantification was performed by calculating the relative mRNA concentration (R0) for each gene/individual sample. Briefly, this was calculated using the following equation: R0 = 1/(1 + E)^Ct where E is the gene efficiency calculated as the average of all individual sample efficiencies across all reactions for a given gene/qPCR plate, and Ct is the cycle number at threshold [71]. The R0 for each gene was normalized to an actin control R0 from each individual sample. Data were tested for normality and differences between means for parasitized and nonparasitized leans and siscowets were analyzed by Student’s *t*-test.

### IPA analysis

Complete sequences obtained from the edgeR analysis were uploaded to Ingenuity Pathway Analysis (IPA) to analyze potential biochemical and physiological pathways that were being regulated in the liver during lamprey parasitism (IPA®, QIAGEN Redwood City, www.qiagen.com/ingenuity). *P*adj values of ≤0.05 were used for all IPA analyses and the significance of potential pathways was analyzed in IPA using the Benjamini-Hochberg method [72] that provides a corrected *p* value to control for the rate of false discovery. The results from edgeR rather than DESeq2 were used since they were conservative in terms of the total number of genes that were regulated but larger than the gene list from the intersection of the DESeq2 and edgeR analyses. Gene pathway names are taken verbatim from IPA and are italicized when referred to in the text.

## Additional files

Additional file 1: Sequences: Number of sequences obtained for each individual sample/treatment and the number of sequences mapped/individual/treatment. (XLSX 51 kb) Additional file 2: Contigs: Full contigs produced by Trinity and used for mapping. (FA 25936 kb) Additional file 3: Lean DESeq2: DESeq2 analysis of transcripts from parasitized versus nonparasitized lean livers. (XLSX 7559 kb) Additional file 4: Lean edgeR: edgeR analysis of transcripts from parasitized versus nonparasitized lean livers. (XLSX 6360 kb) Additional file 5: Siscowet DESeq2: DESeq2 analysis of transcripts from parasitized versus nonparasitized siscowet livers. (XLSX 7727 kb) Additional file 6: Siscowet edgeR: edgeR analysis of transcripts from parasitized versus nonparasitized siscowet livers. (XLSX 6375 kb) Additional file 7: Lean IPA: All IPA pathways compared to the edgeR analysis of parasitized and nonparasitized lean lake trout. (XLSX 90 kb) Additional file 8: Siscowet IPA: All IPA pathways compared to the edgeR analysis of parasitized and nonparasitized siscowet lake trout. (XLSX 103 kb) Additional file 9: QPCR Primers: Forward and reverse primers used for qPCR analysis. (DOCX 112 kb)
