# Supplementary material for: Sea lampreys elicit strong transcriptomic responses in the lake trout liver during parasitism
Source: BMC Genomics. 2016 Aug 24;17(1):675. doi: 10.1186/s12864-016-2959-9 (PMC4997766; doi:10.1186/s12864-016-2959-9)
Supplement: Additional file 9: — QPCR Primers: Forward and reverse primers used for qPCR analysis. (DOCX 112 kb) [file 12864_2016_2959_MOESM9_ESM.docx]

| \| Additional File 9: Gene primers for QPCR. Contig # refers to contigs supplied in Additional File 2. All sequences are 5' to 3' \| \| \| \| \| --- \| --- \| --- \| --- \| \|  \|  \|  \|  \| \|  \|  \|  \|  \| \| Gene Name \| Contig # \| Forward Primer \| Reverse Primer \| \| interleukin-18-binding protein \| comp44889 \| CATCAGCTACCTCTGGGAATG \| TCTAGGAAAGCGGTTGGTTATT \| \| fibulin 4 precursor \| comp81242 \| TGTGTGATCCGGTCCTATCT \| CTTTCTGTAGGTGGCTGTGAA \| \| glucose-6-phosphate isomerase \| comp7122 \| CCAACACTCCCATTAACGTAGA \| CATAAAGTGCGTAGTGGCGA \| \| 6-phosphogluconate dehydrogenase, decarboxylating \| comp4673 \| GCTTTGACCAGCATCACAATC \| TTATCTTGGTGCCTTTAGCCTC \| \| ornithine decarboxylase \| comp4006 \| TATGTGAACGATGGCGTCTATG \| CCAAAGCCAGATGAGCGTA \| \| cyclic AMP-dependent transcription factor ATF-3 \| comp224599 \| GTAGGGTACTACTCACTCACTCA \| TTCACTTGACGCAGGTACAG \| \| indoleamine 2,3-dioxygenase 2 \| comp228271 \| CAGGGAGAATGAAGCCAGAC \| CATGGCCACTACTATCACAGAC \| \| indoleamine 2,3-dioxygenase 2 \| comp275449 \| GGAGGATGACACAGTCAAGATG \| ATGGGTAAGGATGGGAGGAA \| \| adenylosuccinate synthetase isozyme 1 A \| comp122752 \| GATTCCCTATTTCCCAGCTAACA \| GCAAATCTTCCCACTTCCTACA \| \| UDP-glucuronosyltransferase 2A2 \| comp11363 \| GCAAGCCTTTGACAAGAGTT \| GGTAGAGGTTTGGAGGGTTTA \| \| phosphotriesterase related \| comp61354 \| CAAGACAAAGCGACAGTTTCTG \| TCAGAACGGAAAGCACCATATT \| \| aspartoacylase \| comp190057 \| GTTTCGTCTCAACTCCCTTTCT \| CGAGGCTAGAAGAGTTGCTATTT \| \| growth arrest and DNA damage inducible protein \| comp8599 \| ACTCGACACCACCGATAGA \| CTATCTTACGGTGGGAGTCTAC \| \| chemokine-like receptor 1 \| comp22172 \| GGCCTTAACCACAACTCCTT \| CGTCAGGGTAGTCCTCATAGT \| \| dual specificity protein phosphatase 2 \| comp204068 \| TGAGGTAGGCCAGACAGATAG \| TCATAGACTCGGTGAAGGAGAG \| \| ubiquitin carboxyl-terminal hydrolase 24 \| comp72729 \| GAAGATGAGCATCCTGCGATAC \| CGTCAAACACTGCAACCAATAC \| \| DNA damage-regulated autophagy modulator protein 2 \| comp92592 \| GACCAGAAGAAACGGGTTACA \| CTCGGATGTAAGTGAGGAAGAAG \| \| GTP-binding protein Rhes \| comp55581 \| GTCTCCACGTAGCTGTTGTT \| GGTCAAGGAGGACAAGTACAC \| \| C-C motif chemokine 19 \| comp242068 \| CAGCAGCAAACACAGATGAAG \| CTCCACTGACTCTCTGGATACT \| |
| --- | --- | --- | --- | --- | --- | --- | --- | --- | --- | --- | --- | --- | --- | --- | --- | --- | --- | --- | --- | --- | --- | --- | --- | --- | --- | --- | --- | --- | --- | --- | --- | --- | --- | --- | --- | --- | --- | --- | --- | --- | --- | --- | --- | --- | --- | --- | --- | --- | --- | --- | --- | --- | --- | --- | --- | --- | --- | --- | --- | --- | --- | --- | --- | --- | --- | --- | --- | --- | --- | --- | --- | --- | --- | --- | --- | --- | --- | --- | --- | --- | --- | --- | --- | --- | --- | --- | --- | --- | --- | --- | --- | --- |
